# Back-to-Back Performance of the Full Spectrum Nonlinear Fourier Transform and Its Inverse †

**DOI:** 10.3390/e22101131

**Published:** 2020-10-06

**Authors:** Benedikt Leible, Daniel Plabst, Norbert Hanik

**Affiliations:** Institute for Communications Engineering, Technical University of Munich, Theresienstr. 90, 80333 Munich, Germany; daniel.plabst@tum.de (D.P.); norbert.hanik@tum.de (N.H.)

**Keywords:** fiber-optic communications, nonlinear fourier transform, inverse scattering, algorithms

## Abstract

In this paper, data-transmission using the nonlinear Fourier transform for jointly modulated discrete and continuous spectra is investigated. A recent method for purely discrete eigenvalue removal at the detector is extended to signals with additional continuous spectral support. At first, the eigenvalues are sequentially detected and removed from the jointly modulated received signal. After each successful removal, the time-support of the resulting signal for the next iteration can be narrowed, until all eigenvalues are removed. The resulting truncated signal, ideally containing only continuous spectral components, is then recovered by a standard NFT algorithm. Numerical simulations without a fiber channel show that, for jointly modulated discrete and continuous spectra, the mean-squared error between transmitted and received eigenvalues can be reduced using the eigenvalue removal approach, when compared to state-of-the-art detection methods. Additionally, the computational complexity for detection of both spectral components can be decreased when, by the choice of the modulated eigenvalues, the time-support after each removal step can be reduced. Numerical simulations are also carried out for transmission over a Raman-amplified, lossy SSMF channel. The mutual information is approximated and the eigenvalue removal method is shown to result in achievable rate improvements.

## 1. Introduction

In state-of-the-art fiber-optic transmission systems, the achievable information rates (AIR) for high-input powers are limited due to the Kerr nonlinearity of optical fibers [1,2]. This leads to a peak in the AIR curve at some input power, after which the AIR decreases again. To date, it has not been well established if and how the resulting gap between the famous Shannon limit [3] and the decreasing AIR curve for high-input powers can be closed for fiber-optic channels. Due to these inherent rate limitations, current linear modulation schemes, such as wavelength-division multiplexing (WDM), are assumed not to cope with the increasing demand for higher data-rates. This growing demand is largely caused by services like high-resolution, on-demand video streaming and cloud computing [4].

Finding alternatives to the systems in place has been a field of extensive study in recent years. While approaches such as space division multiplexing (SDM) [5,6] can be used to add another dimension along which systems can be scaled to meet future demands, extensive changes in the infrastructure of the underlying communication system have to be made. A number of other approaches such as digital back-propagation (DBP) [7,8,9] and phase conjugated twin waves (PCTW) [10,11] are methods to mitigate nonlinear perturbations by only changing parts of the transmitter and/or receiver, or in the case of mid-span spectral inversion (MSSI) [12,13] adding some functionality, e.g., in the middle of each fiber span. Another approach, which is in the field of study for this publication, uses modulation schemes based on the nonlinear Fourier transform (NFT) [14,15,16,17,18,19,20,21]. While SDM and MSSI have the drawback that the fiber in the communication system would have to be replaced, or at least modified, which would require, e.g., unearthing them, DBP has the drawback that it potentially exhibits high computational complexity, while the PCTW approach, at least in its original implementation, halves the spectral efficiency (SE) of the system, while more advanced PCTW systems still have to sacrifice at least 20% SE [11]. NFT-based transmission systems are looking to reduce computational complexity in comparison to other approaches while also increasing the SE in the high-input power regime [22].

The NFT is a mathematical transformation, representing a time-domain signal by its corresponding nonlinear spectrum. This nonlinear spectrum is often called *nonlinear Fourier spectrum* in an analogy to the behaviour of the standard linear Fourier spectrum for linear channels. The nonlinear Fourier spectrum of a time-domain pulse consists of quantities that are propagating independently according to a simple multiplicative relation for the lossless deterministic nonlinear Schrödinger equation (NLSE) [23,24].

Based on the celebrated work of Zakharov and Shabat [14] from 1972, the vigorous study of mathematical foundations for NFT-based systems already started some decades ago. This mathematical foundation enabled the utilization of fiber optic communications, as shown in the important work by Hasegawa and Nyu [15], where the eigenvalues of simple solitonic pulses were used for data transmission. Several years later, a resurgence in the field of NFT for fiber optic communication lead to a wide variety of publications summarizing prior results and improving algorithms and numerical methods [16,17,18,19,25,26,27]. A lot of new experimental work in this field was published as well [28,29,30,31,32], demonstrating that NFT-aided systems could be built in reality.

Systems utilizing the NFT for modulation, often called nonlinear frequency division multiplexing (NFDM) systems, possess properties which make them beneficial for fiber-optic communications. However, several drawbacks of the scheme became apparent as well: Since the NFT is derived for the lossless deterministic NLSE, further effects occurring in real fiber-optic channels such as loss, amplifier noise and polarization mode dispersion (PMD) lead to distortions in the nonlinear frequency domain. With the NFT being mathematically involved, many of these effects have not yet been fully investigated in the nonlinear Fourier domain. For residual fiber-loss in either the erbium-doped fiber amplifier (EDFA) [23] or distributed Raman amplification (DRA) [33] case, little about the impact on the nonlinear Fourier spectrum is established analytically. However, some mitigation techniques for both cases were published in [34,35,36]. Similarly, the impact of amplification noise of the EDFA and DRA case is not yet fully quantified in the nonlinear Fourier domain. However, some analytic results on the effect of additive white Gaussian noise (AWGN) for simplified cases have already been published [37,38,39,40].

The early algorithms for the NFT and inverse nonlinear Fourier transform (INFT) exhibited numerical instabilities and/or high complexity. Many publications proposed stabilized versions of the transformations and algorithms with reduced complexity [41,42,43,44,45,46,47]. The method presented in [46] is the basis for the studies presented in this publication. The possibility to remove discrete eigenvalues from received pulses is used to improve the full spectrum NFT. This method reduces the numerical error and computational complexity, compared to a state-of-the-art search-based method.

This publication is structured as follows. In Section 2, the necessary preliminaries for the NFT are introduced, giving a short overview of the fundamental ideas. For an in-depth explanation of the mathematical background of the NFT and its inverse, the reader is referred to other publications whenever details are skipped. In Section 3, the used algorithms are presented in detail. This includes an implementation of the full spectrum INFT used for signal generation, the search-based NFT that is used as a bottom-line for comparison, the eigenvalue removal (ER) based full spectrum NFT implementation and a short analysis of the computational complexity of the algorithms. In Section 4, numerical simulation results are presented. Two NFT algorithms are compared regarding normalized mean square error (NMSE) and computation time in a back-to-back transmission scenario. Additionally, the SE of a simplified transmission system is compared for both NFTs presented. In Section 5, the results of Section 4 are discussed, while conclusions and suggestions for future work are given in Section 6.

## 2. The Nonlinear Fourier Transform

The NFT is derived for the lossless, noise-free fiber-optic channel, governed by the lossless and deterministic NLSE. To simplify the following statements, the normalized unitless version of the NLSE is used. Details on the normalization can be found in [16]. The normalized NLSE for anomalous dispersion is given by

(1) $$\frac{\partial q(t,z)}{\partial z}=j\frac{\partial^{2}q\left(t,z\right)}{\partial t^{2}}+j2{\left|q\left(t,z\right)\right|}^{2}q\left(t,z\right),$$

where j is the imaginary unit and q(t,z), *t* and *z* are the normalized slowly varying signal envelope, normalized time and normalized propagation distance, respectively. The NFT/INFT is always carried out for a specific normalized propagation distance *z*, that is constant during transformation. Thus, for the following definition of the nonlinear Fourier spectrum, the normalized propagation distance is assumed to be of constant value, which is identical for the time and nonlinear frequency domain, and thus can be dropped.

The NFT computes the nonlinear Fourier spectrum of a time domain signal, supported on certain parts of the complex valued *nonlinear frequencies* λ. The nonlinear spectrum is commonly divided into two distinct parts, the continuous and discrete nonlinear spectrum, depending on the part of the complex λ plane which they are supported on [16]. The *continuous nonlinear spectrum* is supported along the whole real line (λ∈R) and is given by

(2) $$q_{c}\left(\lambda \right)=\frac{b(\lambda )}{a(\lambda )}\;\;\mathrm{with}\;\;\lambda \in R,$$

where a(λ) and b(λ) are called the *nonlinear Fourier coefficients*. For details on the nonlinear Fourier coefficients, we refer the reader to [16,17]. The values b(λ) can also be modulated directly, which has been shown to be beneficial compared to modulating the nonlinear spectral amplitudes $q_{c}\left(\lambda \right)$ [48,49,50,51]. The continuous spectrum can be viewed in analogy to the linear frequency spectrum, obtained by the standard linear Fourier transform (FT). The frequency components of the standard linear FT propagate decoupled in a convolutional channel. Similarly, for the channel governed by Equation (1), the spectral components of the continuous nonlinear Fourier spectrum propagate decoupled. This also holds for the discrete nonlinear spectral components, which are introduced in the following.

The *discrete nonlinear spectrum* is only supported on a finite number *K* of nonlinear frequencies $\lambda_{k}$ in the positive complex half plane $\left(\lambda_{k}\in C^{+}\right)$. Those discrete points, satisfying $a(\lambda_{k})=0$, are given by [16]

(3) $$q_{d}\left(\lambda_{k}\right)=\frac{b(\lambda_{k})}{a^{\prime }\left(\lambda_{k}\right)}\;\mathrm{with}\;a^{\prime }\left(\lambda \right)=\frac{da(\lambda )}{d\lambda }.$$

For the discrete spectrum, the values $b(\lambda_{k})$ can also be modulated directly [52,53]. While the NFT has a lot of interesting and useful properties [16,19], we highlight just two properties, which will be used again later. First an energy relation, similar to Parseval’s theorem, gives a direct relation between the time-domain energy and the energy in the respective continuous and discrete nonlinear spectrum. The energies can be computed according to [16]

(4) $$\begin{array}{ccc} E_{t} & = & \int_{-\infty }^{\infty }{\left|q\left(t\right)\right|}^{2}dt=E_{c}+E_{d}, \end{array}$$

(5) $$\begin{array}{ccc} E_{c} & = & \frac{1}{\pi }\int_{-\infty }^{\infty }\log(1+|q_{c}\left(\lambda \right){|}^{2})d\lambda , \end{array}$$

(6) $$\begin{array}{ccc} E_{d} & = & 4\sum_{k=1}^{K}ℑ\left(\lambda_{k}\right), \end{array}$$

where $E_{t}$ is the pulse energy computed in the time domain and $E_{c}$, $E_{d}$ is the energy carried in the continuous and discrete nonlinear spectrum of the pulse, respectively. Note that the energy in the discrete spectrum only depends on the imaginary part of the discrete eigenvalues $\lambda_{k}$.

The second important property is the *Lax convolution* property [16]. Since we now deal with spectra obtained at different normalized propagation distances, the distance parameter *z* is reintroduced. In the following, we assume that the pulse was transformed by the NFT for the first time at z=0 and transformed a second time after propagating for a normalized distance $z=L_{n}$ over the optical channel, given by Equation (1).

Even though the time domain pulse is distorted by the complicated interaction of dispersion and nonlinearity during propagation along the fiber, the nonlinear spectrum changes according to simple relations. The nonlinear frequencies remain constant during propagation and the nonlinear spectral components change according to [16]

(7) $$\lambda \left(0\right)=\lambda \left(L_{n}\right),\;\;\;\frac{q_{c}\left(\lambda ,L_{n}\right)}{q_{c}\left(\lambda ,0\right)}=e^{4j\lambda^{2}L_{n}},\;\;\;\frac{q_{d}\left(\lambda_{k},L_{n}\right)}{q_{d}\left(\lambda_{k},0\right)}=e^{4j\lambda_{k}^{2}L_{n}}.$$

As stated previously, the spectral components propagate independently for each nonlinear frequency λ, similar to frequency components in the conventional frequency domain, propagating independently in convolutional channels.

## 3. Algorithms

The nonlinear Fourier spectrum of a signal can only be calculated analytically for a small number of special pulse shapes [16,54]. Thus, the NFT/INFT has to be computed numerically for most signals that are relevant for data transmission. In transmission systems modulating the nonlinear Fourier spectrum of a pulse, the INFT is used at the transmitter to compute the corresponding time-domain pulse. This pulse can then be transmitted over the fiber channel of normalized length $L_{n}$, subject to dispersion, nonlinearity, fiber-loss and added noise. At the receiver, the NFT is then used to calculate the nonlinear Fourier spectrum of the received pulse. If the channel is ideal in the sense that it is governed by Equation (1) and the NFT/INFT are assumed to be exact, the resulting nonlinear spectrum at the receiver ($q_{c}\left(\lambda ,L_{n}\right),\left\{\lambda_{k},q_{d}\left(\lambda_{k},L_{n}\right)\right\}$) and the modulated spectrum at the transmitter ($q_{c}\left(\lambda ,0\right),\left\{\lambda_{k},q_{d}\left(\lambda_{k},0\right)\right\}$) fulfill Equation (7). However, since the transformations are computed numerically, certain numerical errors are introduced. To minimize numerical errors for both INFT and NFT, many different algorithms and implementations are known in the literature [17,18,19,20,21,27,41,43,44,45,54,55,56,57,58,59].

In the following section, three algorithms are presented. In Section 3.1, the INFT algorithm to transform the modulated nonlinear Fourier spectrum is presented. Its implementation was carried out according to [60]. For recovering the nonlinear Fourier spectrum at the receiver, a variety of algorithms were proposed. The majority of these algorithms split the process into continuous and discrete transformation. For many of the continuous spectrum NFTs the algorithms differ only in the used integration scheme. The discrete spectrum is often obtained either by search or matrix-eigenvalue methods [17]. However there are also some promising recent results utilizing the evaluation of contour integrals [54] or tracking the phase jump of a(λ) [55]. Since, for most practical cases, search-based methods exhibit lower computational complexity than matrix-eigenvalue based methods and a search-based method is used in [46], on which our further studies are based, a search-based method is presented in Section 3.2 for comparative purposes. In Section 3.3 the ER-aided NFT is presented, which is a modification of the previously mentioned search-based NFT. This method is based on [46] and is extended for additional detection of the continuous spectrum.

### 3.1. The Full Spectrum Inverse Nonlinear Fourier Transform

**Figure 1:**
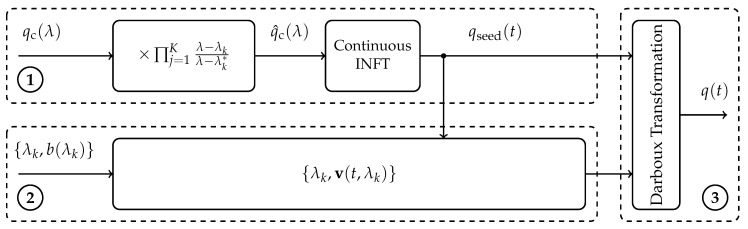
Block diagram of joint spectrum modulation (based on [60]).

Depending on the properties of the spectrum that is to be converted into a time-domain pulse, different algorithms for the INFT are known. If the nonlinear spectrum is purely continuous, special INFT algorithms exist for its transformation [17,20]. For purely discrete spectra ($q_{c}\left(\lambda \right)=0$), the Darboux transform (DT) is a widely adopted scheme to generate multi-solitons [18,19]. However, to fully utilize the nonlinear Fourier spectrum, both spectral components should be used in a full spectrum INFT [28,60]. Figure 1 shows the building blocks of this joint INFT. It can be seen that this full spectrum INFT utilizes distinct INFTs for certain parts of the spectrum.

In the upper branch labeled ①, an initial seed solution $q_{\mathrm{seed}}\left(t\right)$ is generated from the continuous part $q_{c}\left(\lambda \right)$ of the modulated nonlinear spectrum. Note that $q_{c}\left(\lambda \right)$ is predistorted before the continuous INFT step, generating ${\hat{q}}_{c}\left(\lambda \right)$. This is necessary, since the continuous spectrum of a pulse changes according to [19]

(8) $$q_{c}\left(\lambda ;\lambda_{0}\right)=\frac{\lambda -\lambda_{0}^{*}}{\lambda -\lambda_{0}}q_{c}\left(\lambda \right),$$

when the discrete eigenvalue $\lambda_{0}$ is added to the time domain pulse $q_{\mathrm{seed}}\left(t\right)$ using the DT. As a result, to get $q_{c}\left(\lambda \right)$ to be the continuous spectrum of q(t) at the end of the DT in ③, the continuous spectrum of $q_{\mathrm{seed}}\left(t\right)$ has to be ${\hat{q}}_{c}\left(\lambda \right)$. The continuous INFT was implemented by the inverse Ablowitz–Ladik scheme [20], but, in general, any continuous INFT method can be used.

**Figure 2:**
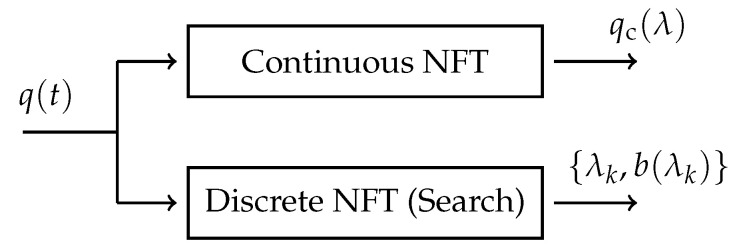
Block diagram of the search-based NFT.

The DT in part ③ of Figure 1, is used to iteratively add discrete eigenvalues and their corresponding discrete b-values to the seed solution $q_{\mathrm{seed}}\left(t\right)$. Note that we modulate b-values directly. For an in-depth explanation of the DT, we refer the reader to [19]. Since the modulated b-values $b(\lambda_{k})$ cannot be directly forwarded to the DT, the appropriate parameters $v(t,\lambda_{k})$ have to be calculated for the generated seed pulse $q_{\mathrm{seed}}\left(t\right)$ (Figure 1, ②). There are many ways to compute these values utilizing the Zakharov-Shabat (ZS)-system [14], however we found utilizing a modified version of the forward backward method [19] which can be found in [60] to work well. Before we conclude this section, we would like to point out that the presented method is not the only way to implement an INFT for continuous and discrete spectrum pulses. Some other applicable methods can be found in [56,57,58,59].

### 3.2. The Search-Based Nonlinear Fourier Transform

A search-based NFT was implemented to compare the eigenvalue removal method, described in Section 3.3, to a state-of-the-art NFT. As previously mentioned, for the search-based approach, the continuous and discrete nonlinear Fourier spectra are obtained separately from the received pulse q(t). Figure 2 shows the block diagram of the NFT.

For the continuous spectral part, the layer peeling NFT is used [17]. Layer peeling was chosen as it leads to good numerical results and is computationally cheap, compared to other methods [17]. For the discrete spectrum, a search-based algorithm was used. Search-based algorithms start at initial guesses for a discrete eigenvalue and then by [17]

(9) $$\lambda_{\left(i+1\right)}=\lambda_{\left(i\right)}-\alpha_{d}\frac{a(\lambda_{\left(i\right)})}{a^{\prime }\left(\lambda_{\left(i\right)}\right)}\;,$$

iteratively search for the zeros of a(λ) in the positive complex half-plane. Here, the subscript (i) denotes the search iteration and the parameter $\alpha_{d}$ is the dampening factor that can be used to scale the stepsize of the search. In this publication, the dampening factor is set to $\alpha_{d}=1$. The search is stopped if $|\alpha_{d}\frac{a(\lambda_{(}i))}{a^{\prime }\left(\lambda_{i}\right)}|<\delta_{\mathrm{search}}$ for a sufficiently small $\delta_{\mathrm{search}}$. Using the estimate for the discrete eigenvalue $\lambda_{k}$, the corresponding b-value $b(\lambda_{k})$ is obtained. The nonlinear Fourier coefficients a(λ),b(λ) and derivative $a^{\prime }\left(\lambda \right)$ are computed using the forward–backward method, as described in [19].

Note that in recent publications [54,55], two interesting methods utilizing contour integral evaluation and phase jump tracking of a(λ) are presented, respectively. Additionally, [54] also compares a wide variety of existing algorithms regarding their accuracy.

### 3.3. The Eigenvalue Removal Nonlinear Fourier Transform

To increase the reliability at the detection side, a recently presented method to remove discrete eigenvalues from a received time-domain pulse is used [46]. This method was introduced in the context of multi-soliton pulses with a purely discrete nonlinear spectrum. We extend its use to pulses with additional continuous spectrum components.

**Figure 3:**
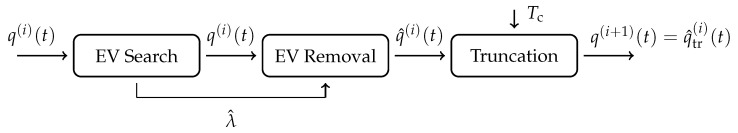
Block diagram for one eigenvalue removal step.

The detection of the discrete nonlinear spectrum is still based on the search method, described in Section 3.2. However, instead of searching for the whole discrete spectrum at once, the time-domain pulse is modified after each search iteration. A block diagram of one of these augmented search iterations is shown in Figure 3.

At first, the search algorithm from Section 3.2 is used to estimate the discrete eigenvalue $\hat{\lambda }$ with the smallest imaginary part. Subsequently, this discrete eigenvalue is removed from the discrete spectrum of the pulse, yielding the altered pulse ${\hat{q}}^{\left(i\right)}\left(t\right)$. After the successful removal of $\hat{\lambda }$, the pulse is assumed to be more compact in time [61] and its support can, therefore, be truncated so that, e.g., 99.9% of the pulse energy is contained in the truncation interval $\left[t_{1}^{\left(i\right)},t_{2}^{\left(i\right)}\right]$. This results in a reduced amount of samples that have to be processed in the next step. For the next iteration (i+1), the search algorithm is used with the truncated pulse $q^{\left(i+1\right)}\left(t\right)={\hat{q}}_{\mathrm{tr}}^{\left(i\right)}\left(t\right)$ and thus, the numerical error as well as the complexity is reduced in comparison to the search-based NFT described in Section 3.2 [46]. For the initial step i=1, the received pulse is used as the input $q^{\left(i=1\right)}\left(t\right)=q\left(t\right)$. At the end of this section, we discuss the benefit of processing the discrete eigenvalues in the order of increasing imaginary parts.

**Figure 4:**
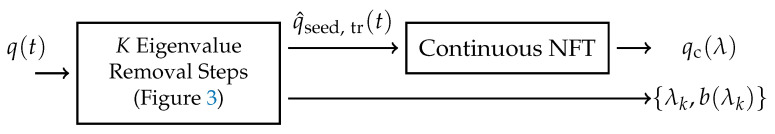
Block diagram of the ER NFT.

The search method from Section 3.2 can be used without changes, reducing the initial guesses to the eigenvalue with the smallest imaginary part remaining in the pulse. The estimated discrete eigenvalue $\hat{\lambda }$ and the corresponding b-value $b(\hat{\lambda })$ are stored and $\hat{\lambda }$ and $q^{\left(i\right)}\left(t\right)$ are forwarded to the eigenvalue removal block. Here, the relation [46]

(10) $${\hat{q}}^{\left(i\right)}\left(t\right)=q^{\left(i\right)}\left(t\right)+\frac{2j\left({\hat{\lambda }}^{*}-\hat{\lambda }\right)v_{2}^{*}\left(t,\hat{\lambda }\right)v_{1}\left(t,\hat{\lambda }\right)}{|v_{1}\left(t,\hat{\lambda }\right){|}^{2}+{\left|v_{2}\left(t,\hat{\lambda }\right)\right|}^{2}}\;\mathrm{with}\;\left(\begin{array}{c} cv_{1}\left(t,\hat{\lambda }\right)\\ v_{2}\left(t,\hat{\lambda }\right) \end{array}\right)=v\left(t,\hat{\lambda }\right),$$

is used to remove the estimated eigenvalue $\hat{\lambda }$ from the time-domain pulse. $v(t,\hat{\lambda })$ can be obtained from $q^{\left(i\right)}\left(t\right)$, as mentioned in Section 3.1. Note that this step can fail if the difference between the estimated discrete eigenvalue $\hat{\lambda }$ and the true value in the discrete spectrum of $q^{\left(i\right)}\left(t\right)$ is too large. In this case, the discrete spectrum would be augmented by adding $\hat{\lambda }$ to the discrete spectrum of $q^{\left(i\right)}\left(t\right)$. The success of this step can be verified by monitoring the pulse energy. If the removal was successful, the energy should have decreased by $\approx 4ℑ(\hat{\lambda })$, compared to the energy of $q^{\left(i\right)}\left(t\right)$ (see (4)) and the pulse is truncated to the interval $\left[t_{1}^{\left(i\right)},t_{2}^{\left(i\right)}\right]$. In case of an unsuccessful removal, the altered pulse ${\hat{q}}^{\left(i\right)}\left(t\right)$ is discarded and $q^{\left(i+1\right)}\left(t\right)=q^{\left(i\right)}\left(t\right)$ is used again in the next search iteration. If the removal step always fails, this method defaults to the search method described in Section 3.2, adding a slight computational overhead for the unsuccessful removal steps. Assuming that the algorithm can remove the discrete spectrum completely from the pulse q(t), the remaining pulse ${\hat{q}}_{\mathrm{seed}},\mathrm{tr}\left(t\right)$ should only contain continuous spectrum components and thus should be similar to a truncated version of $q_{\mathrm{seed}}\left(t\right)$ propagated according to Equation (1). The continuous spectrum can then be recovered from this pulse by any continuous NFT algorithm. We used layer peeling [17], which was also used for the search-based method in Section 3.2. Since the continuous spectrum might bear significantly less energy than the discrete spectrum, the accuracy of the continuous spectrum recovery might be diminished by the truncation steps in the removal process cutting of features of the pulse that are relevant for the continuous spectrum of the pulse. To resolve this problem, a time duration $T_{c}$ is introduced as a lower bound for the truncation interval $\left(t_{2}^{\left(i\right)}-t_{1}^{\left(i\right)}\geq T_{c}\right)$. Although this limits complexity reduction, it preserves the continuous spectrum. Note also that by successful removal of an eigenvalue, the continuous spectrum is altered according to the inverse of Equation (8), which has to be taken into account after the transformation. The full eigenvalue removal NFT algorithm is also depicted in Figure 4.

**Figure 5:**
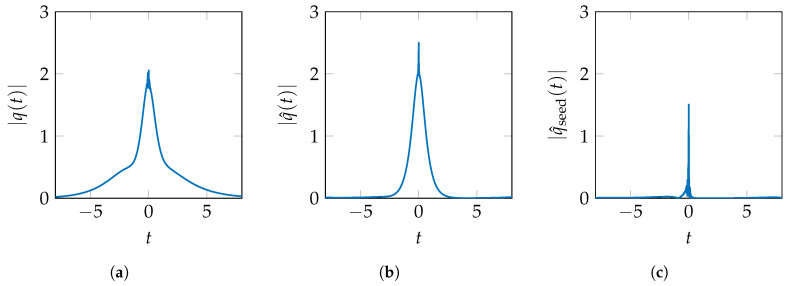
Altered pulses in the eigenvalue removal NFT. (**a**) Initial pulse *q*(*t*) with continuous and discrete spectrum (**b**) After removal of λ_1_ = j0.25 (**c**) After also removing λ_2_ = j (only continuous spectrum remains).

To motivate the ordering for the removal steps, one has to look at the shape of solitons with one discrete eigenvalue. It can be shown that the time interval, confining, e.g., 99.9% of the pulse energy, is inversely proportional to the imaginary part of the discrete eigenvalue [62]. While it is true that multi-solitons cannot be constructed by linear superposition of simple solitons, in many cases, features of the basic soliton shapes, corresponding to the discrete eigenvalues, are still visible in the multi-soliton pulse. This observations and the findings in [61] on the behaviour of symmetric multi-soliton pulses for |t|→∞ lead us to remove eigenvalues in the order of increasing imaginary parts, potentially removing temporally broad features of the pulse first. This enables a stronger truncation of the altered time domain pulse in the first iterations. An example of this deconstruction, for a pulse with two discrete eigenvalues and a nontrivial $q_{\mathrm{seed}}\left(t\right)\neq 0$, is given in Figure 5 without truncation.

The nonlinear spectrum of the initial pulse in Figure 5a consists of the continuous spectrum $q_{c}$, the discrete eigenvalues $\lambda_{1}=j0.25,\lambda_{2}=j$ and corresponding b-values $b\left(\lambda_{1}\right)=1,b\left(\lambda_{2}\right)=j$. In Figure 5b, the discrete eigenvalue with the smallest imaginary part has been removed. The continuous spectrum is still present in the pulse but is now distorted according to the inverse relation shown in (8). The discrete spectrum includes only the remaining $\lambda_{2}=j$ and its corresponding b-value. In Figure 5c, the second discrete eigenvalue $\lambda_{2}$ has also been removed, further distorting the continuous spectrum according to (8). This pulse is similar to the initial seed pulse $q_{\mathrm{seed}}\left(t\right)$ after proper mitigation of the mentioned distortions in $q_{c}\left(\lambda \right)$ from the removal process and the propagation according to Equation (1).

### 3.4. Complexity of the NFT Algorithms

The complexity of the discussed NFT algorithms depends on several factors. In addition to the number of samples *N* for the time domain pulse q(t), the specific algorithm for each calculation of the nonlinear Fourier coefficients a(λ), b(λ) factors into the overall complexity. To obtain the nonlinear Fourier coefficients for a nonlinear frequency λ, O(N) multiplications and additions have to be executed [17]. As a result, the complexity of the continuous spectrum NFT is given by $O(N^{2})$ operations, assuming the continuous spectrum is calculated on a *N*-point grid [17].

The complexity of one search iteration of the discrete search-based method, described in Section 3.2, is given by O(N). The overall complexity also depends on the number of discrete eigenvalues *K* and the number of search iterations for each discrete eigenvalue, which depends on the channel perturbations, the transmitted pulse shape, the dampening factor $\alpha_{d}$ and the initial guesses $\lambda_{\left(0\right)}$ (see (9)).

For the ER algorithm, the complexity for one search iteration can be expressed by $O(\alpha_{t}\left(\lambda_{k}\right)N)$, where $\alpha_{t}\left(\lambda_{k}\right)$ with k∈{1,⋯,K} reduces the amount of necessary samples prior to starting the search procedure. The overall complexity also depends on the number of discrete eigenvalues *K* and the number of search iterations for each discrete eigenvalue. For a complexity comparison of the search-based and the ER-NFT, the number of search steps can be assumed as approximately equal, since the same received pulse q(t) is evaluated. As described in Section 3.3, truncation is limited by $T_{c}$, and thus $\alpha_{t}\left(\lambda_{k}\right)\in \left[T_{c}/T_{\mathrm{supp}},1\right]$. It also can be seen that if $\alpha_{t}\left(\lambda_{k}\right)=1\;\forall \;k\in \left\{1,\cdots ,K\right\}$ the complexity of all search steps is O(N), and thus the overall complexity for the discrete spectrum NFT then matches the overall complexity of the search-based method discrete spectrum NFT. The complexity of the continuous spectrum NFT is reduced if the truncated pulse ${\hat{q}}_{\mathrm{seed}},\;\mathrm{tr}\left(t\right)$ is used as shown in Figure 4. The complexity is then given by $O(\alpha_{t}\left(\lambda_{K}\right)N^{2})$. Additionally we would like to note that, since the continuous NFT can be implemented using any algorithm, fast NFT approaches as described in [63] are applicable, potentially reducing the complexity to $O(\alpha_{t}\left(\lambda_{K}\right)N\log^{2}\left(\alpha_{t}\left(\lambda_{K}\right)N\right))$. Since the values for $\alpha_{t}\left(\lambda_{k}\right)$ depend on the specific shape of the received pulse and the width of the pulse in time domain which cannot be linked to the nonlinear Fourier spectrum analytically for K>1, the complexity of the two algorithms from Section 3.2 and Section 3.3 can only be compared in more detail by evaluating the values $\alpha_{t}\left(\lambda_{k}\right)$ numerically for specific cases of interest.

## 4. Simulation Results

**Figure 6:**
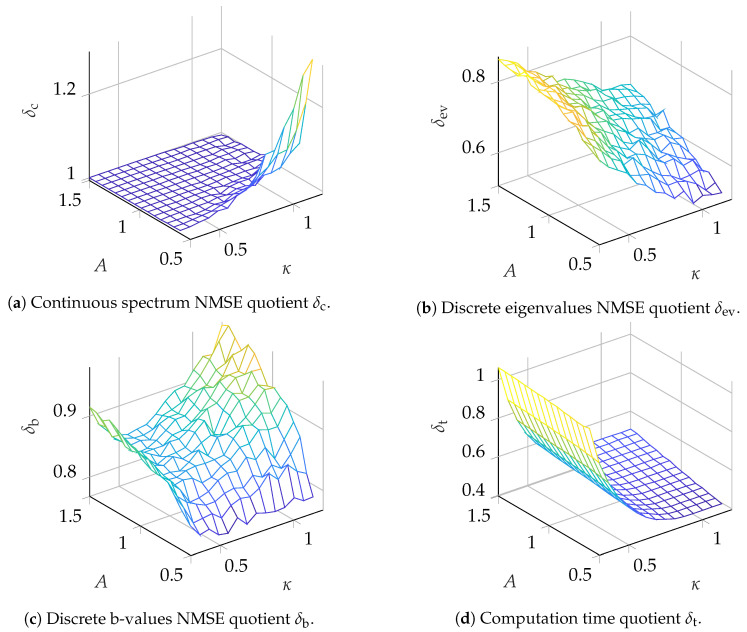
Comparison of detection algorithms for K=2 eigenvalues.

**Figure 7:**
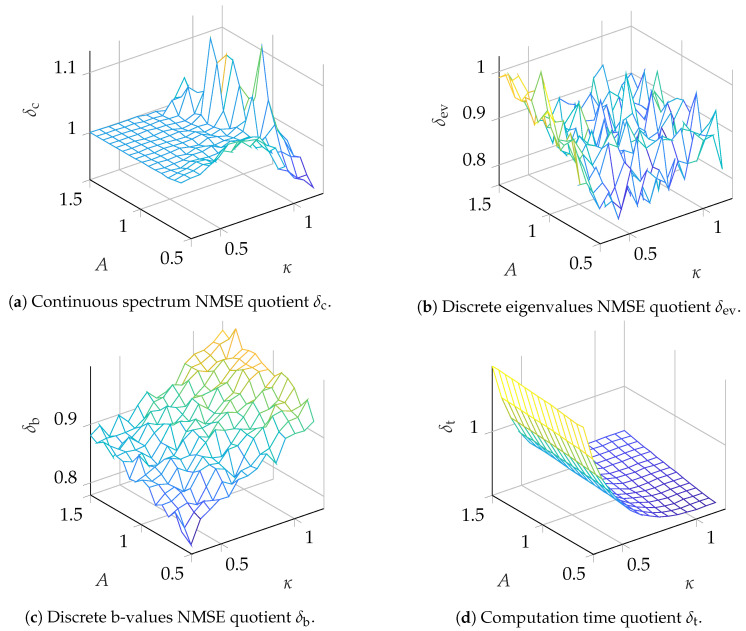
Comparison of detection algorithms for K=3 eigenvalues.

Simulations testing the INFT/NFT configuration in a back-to-back setup were conducted for pulses with $N=2^{10}$ samples. For the continuous spectrum, one nonlinear channel was modulated. The continuous nonlinear frequency support was set according to Equation (22f) in [20]. The channel itself was chosen to be a root raised cosine (RRC) spectrum centered around λ=0 with a nonlinear spectral width of $W_{\lambda }=14.2857$ and a roll-off factor of β=0.15. The channel was amplified to have amplitude *A* in the nonlinear frequency domain. The parameter *A* was changed during simulation. This channel was modulated with a phase shift keying (PSK) symbol $s_{c}$ with i.i.d. uniform random phase ϕ∈[0,2π). The discrete spectrum was modulated in two different ways. For the results depicted in Figure 6, K=2 discrete eigenvalues $(\lambda_{1}=j\kappa ,$$\lambda_{2}=j2\kappa )$ were added to the seed pulse. The corresponding b-values were again modulated with an PSK constellation with i.i.d. uniform random phase ϕ∈[0,2π). For the results depicted in Figure 7, K=3 discrete eigenvalues $(\lambda_{1}=j\kappa ,$$\lambda_{2}=j2\kappa ,$$\lambda_{3}=j3\kappa )$ were added and the corresponding b-values were modulated in the same way as for K=2. The parameter κ was changed during simulation.

Transmission pulses were generated from their respective nonlinear Fourier spectra, using the INFT algorithm presented in Section 3.1. The spectra of the generated pulses were converted back into the nonlinear Fourier domain by both NFTs presented in Section 3.2 and Section 3.3.

For comparison of the two NFT algorithms, the NMSEs between the modulated values ($s_{c}$, $b=[b\left(\lambda_{1}\right),\cdots ,b\left(\lambda_{K}\right)]$) and recovered values (${\tilde{s}}_{c}$, $\tilde{b}=\left[\tilde{b}\left(\lambda_{1}\right),\cdots ,\tilde{b}\left(\lambda_{K}\right)\right]$) were computed. The NMSE for the discrete eigenvalues $\lambda =[\lambda_{1},\cdots ,\lambda_{K}]$ was measured. The NMSEs are denoted as ${\mathrm{NMSE}}_{x}\left(s_{c}\right)$ with x∈{r,s} for the NMSE calculated for continuous spectrum symbols, with the subscript s indicating that the search-based NFT was used and similarly, r indicating that the ER-NFT was used. The NMSE for the discrete eigenvalues is denoted as ${\mathrm{NMSE}}_{x}\left(\lambda \right)$ with x∈{r,s} and the NMSE for the b-values is denoted as ${\mathrm{NMSE}}_{x}\left(b\right)$ with x∈{r,s}. The minimum and maximum values obtained in the simulations are given in Table 1.

To visualize the improvement in the eigenvalue removal NFT over the search-based NFT, the ratio between the NMSEs for both NFTs is computed according to $\delta_{x}={\mathrm{NMSE}}_{r}\left(\cdot \right)/{\mathrm{NMSE}}_{s}\left(\cdot \right)$ with x∈{c,ev,b}. Suffixes c, ev and b denote the ratio of NMSEs for the continuous spectrum symbols, discrete eigenvalues and b-values, respectively. Additionally, the average time used for execution was measured and is given as ratio $\delta_{t}=t_{r}/t_{s}$, where $t_{r}$ is the average execution time for the ER-NFT from Section 3.3 and $t_{s}$ is the average execution time for the search-based NFT from Section 3.2. The results for the scenarios K=2 and K=3 are depicted in Figure 6 and Figure 7, respectively, and are discussed in detail in Section 5. The ranges for the two parameters, which were changed during simulation to obtain the datapoints, are A∈(0.6,1.5) and κ∈(0.3,1.2).

**Figure 8:**
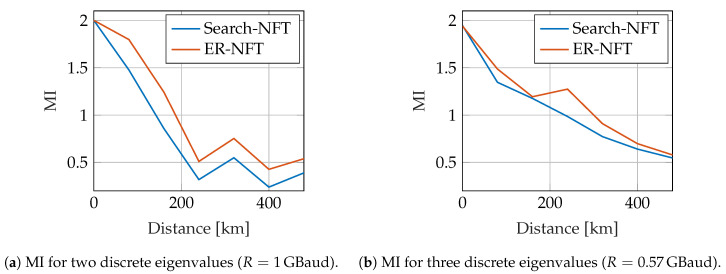
Comparison of NFT algorithms for Raman amplified fiber.

Additionally, data transmission over Raman-amplified fiber was simulated, comparing detection with the search-based NFT and the ER-NFT. Due to the results presented in Figure 6 and Figure 7 the continuous spectrum for the ER-NFT algorithm was computed from the initially received pulse, resulting in identical detection for the continuous spectrum part in both NFT algorithms. The fiber was assumed to be a standard single-mode fiber with α=0.2dB/km, $\beta_{2}=-21.683\;{\mathrm{ps}}^{2}/\mathrm{km}$ and $\gamma =1.3\;W^{-1}\;{\mathrm{km}}^{-1}$. Raman-amplification was implemented with a co- and counterpropagating pump scheme, described in detail in [64]. The total transmission distance was chosen to be 480km with a spanlength of $L_{s}=80\;\mathrm{km}$. The continuous spectrum was modulated using 4-PSK constellation points and the previously described configuration with only one channel in the continuous spectrum. For the discrete spectrum, the b-values of discrete eigenvalues were modulated according to a 4-PSK constellation. The discrete eigenvalues were chosen identically to the back-to-back simulations with κ=1. The pulses were generated with a normalized time duration of $T_{n}=12$ with 1024 samples per pulse and were transmitted with the symbol rates given in Figure 8a,b. In this simulation, pulses were transmitted independently, assuming enough guard time between pulses to ensure independent propagation. Each constellation point was transmitted 100 times for each continuous channel and each discrete eigenvalue. To evaluate the performance of the NFTs, a lower bound of the mutual information (MI) according to [65] was computed after each fiber span. The values presented in Figure 8 are the mean of the MIs that were computed separately for the continuous spectrum and each discrete eigenvalue.

## 5. Discussion

From the results presented in Section 4, it can be seen that the benefit of utilizing the ER-NFT strongly depends on the parameter range used for modulation. Starting with the discussion of the effect on the accuracy of the continuous spectrum NFT, it can be seen from Figure 6a and Figure 7a that for almost the whole range of value pairs tested, the ER-NFT is not beneficial compared to the search-based approach. For large κ, there is even a decrease in accuracy from using the ER-NFT. A small improvement can be seen in Figure 7a for high κ and small *A* values, but the benefit is small and confined. The results imply that the originally received pulse should preferably be used for the continuous NFT instead of the altered pulse ${\hat{q}}_{\mathrm{seed}},\;\mathrm{tr}\left(t\right)$ (compare Figure 4). This would increase the computational complexity of the continuous NFT from $O(\alpha_{t}\left(\lambda_{K}\right)N^{2})$ to $O(N^{2})$, as described in Section 3.4, since the received pulse is not truncated prior to the NFT. On the other hand, this decouples the continuous NFT from the ER step, enabling parallelization, which might again improve computation times on parallel computation architectures.

For the discrete eigenvalues and corresponding b-values, the eigenvalue removal NFT improves the precision for all tested parameter pairs (see Figure 6b,c and Figure 7b,c). For the scenario with K=2, the error for high κ and small *A* values can be reduced by up to ≈50%, while for small κ and big *A* values there is still an improvement of ≈14%. For the b-values, there seems to be an optimum value for κ in the tested region, while the improvement increases for small *A*. Since for small *A* the NMSE for the continuous spectrum reaches its minimum, it could be assessed in future work whether an optimum for *A* can be found in the region A<0.6. For the scenario K=3, depicted in Figure 7, the benefit is not immediately evident. For the discrete eigenvalues (Figure 7b) the algorithm still shows some improvement, but there is no clear slope visible. One possible explanation could be that, for K=3, the discrete eigenvalues have a larger imaginary part and therefore, the limit for the truncation interval $T_{c}$ could be reached more quickly (compare motivation for ”small imaginary part first” ordering in Section 3.3). As a result, the numerical error cannot be reduced further by truncation. Further, since $T_{c}$ poses an ultimate threshold for the reduction in numerical complexity, the current implementations complexity will be very close to the complexity of the search-based method if $T_{c}$ is very close to the temporal width of the received pulse. Since this might limit the usefulness of the ER-NFT to systems with a certain parameter set, this should be investigated further in the future. In Figure 7c, one can see that, for the b-values, the improvement is reduced for larger κ and *A*. We assume that the reason for this behaviour is the less accurate detection of large discrete eigenvalues, which might, in turn, cause larger errors in the eigenvalue removal step or even fail to remove all eigenvalues from the pulse. As discussed earlier in Section 3.3, the algorithm defaults to the accuracy of the search-based NFT if all eigenvalue removal steps fail. Finally, the computation times of both algorithms can be compared (see Figure 6d and Figure 7d). It can be seen that as long as κ is large enough that the algorithm can sufficiently truncate the pulse in each step, the eigenvalue removal NFT reduces the computation time. It can also be seen that the parameter *A* does not influence the computation time in the tested region. For K=2, a maximum reduction of over 60% can be achieved, while for K=3, still a maximum reduction of ≈50% is visible. To ensure a fair comparison, the algorithms were tested on the same hardware under similar conditions. We also deem the eigenvalue removal implementation was less optimized, and assume that by further optimization, its computation time could be reduced even further.

Additionally, in Figure 8, the MI was evaluated over the transmission distance. It can be seen that the achievable rates are improving, if the ER-NFT is used instead of the search-based NFT. As discussed previously in Section 4, the continuous spectrum was detected using the initially received pulse. The gain in the depicted MIs thus solely stems from the improved detection of constellation points transmitted in the discrete spectrum.

Note that several other parameters might influence the accuracy and computation time of the presented algorithms. In the presented simulations, purely imaginary eigenvalues were chosen, which do not move from their allocated time slot during propagation. This changes if the discrete eigenvalues have some nonzero real component. Parts of the pulse corresponding to these eigenvalues might move towards the edges of the time window, and thus might restrict the possible amount of truncation. Another important factor is the spreading of the signal part corresponding to the continuous spectrum. If, due to higher transmission distances, this part of the pulse is spreading, the truncation threshold $T_{c}$ has to be adapted, which might also restrict the amount of truncation possible. Similarly, if the temporal width of the signal part representing the continuous spectrum is depending on the exact way the continuous spectrum is modulated and thus certain parameter sets might also increase the threshold parameter $T_{c}$, reducing the amount of truncation that can be achieved. All these points should be addressed in future studies to assess the applicability of the scheme for more realistic scenarios. The parallel approach for the ER algorithm mentioned in Section 4 should also be considered in future studies. If the reduced complexity of the continuous NFT part is sacrificed for the possibility to parallelize the two steps, it should be evaluated how truncating the received signal beyond the threshold $T_{c}$ influences the accuracy of the discrete spectrum ER-NFT removal steps and if, possibly, complexity can be further reduced that way.

## 6. Conclusions

One major issue regarding NFT-based, fiber-optic systems are the high-complexity algorithms and numerical instabilities when the modulated parameters exceed a certain range of values. To mitigate this effect and reduce the complexity of the algorithms in the receiver for the full nonlinear spectrum transmission, an ER-NFT was implemented. It was shown by simulation that, depending on the used parameters, the proposed NFT shows an improvement in both numerical errors and computation time for the studied system. In future work, other cases, such as discrete eigenvalues with nonzero real part and fully loaded continuous spectrum signals should be investigated to examine the algorithms’ applicability to those cases. Furthermore, optimum parameter regions should be determined. First and foremost, the algorithm should be tested for systems with more realistic parameter sets regarding fiber length and the number of modulated nonlinear frequencies to study the region in which the algorithm improves the receivers of more realistic systems in terms of accuracy and speed.

## Figures and Tables

**Table 1:** Minimum and maximum NMSEs of three spectral parameters for two tested NFTs in two scenarios (K=2,K=3).

	Continuous Spectrum		Discrete Eigenvalues		b-Values	
	min	max	min	max	min	max
${\mathrm{NMSE}}_{s}$ (K=2)	$5.3\times 10^{-5}$	$1\times 10^{-2}$	$5\times 10^{-7}$	$4\times 10^{-4}$	$6.2\times 10^{-7}$	$7.9\times 10^{-5}$
${\mathrm{NMSE}}_{r}$ (K=2)	$5.3\times 10^{-5}$	$1\times 10^{-2}$	$3.8\times 10^{-7}$	$2\times 10^{-4}$	$5.2\times 10^{-7}$	$7.6\times 10^{-5}$
${\mathrm{NMSE}}_{s}$ (K=3)	$5.3\times 10^{-5}$	$1\times 10^{-2}$	$3.5\times 10^{-6}$	$2.9\times 10^{-3}$	$5.2\times 10^{-6}$	$1\times 10^{-3}$
${\mathrm{NMSE}}_{r}$ (K=3)	$5.3\times 10^{-5}$	$1\times 10^{-2}$	$3\times 10^{-6}$	$2.7\times 10^{-3}$	$4.2\times 10^{-6}$	$1\times 10^{-3}$

## Abbreviations

The following abbreviations are used in this manuscript:

AIR	Achievable Information Rates	NFT	Nonlinear Fourier Transform
AWGN	Additive White Gaussian Noise	NLSE	Nonlinear Schrödinger Equation
DBP	Digital Back-Propagation	NMSE	Normalized Mean Square Error
DRA	Distributed Raman Amplification	PCTW	Phase Conjugated Twin Waves
DT	Darboux Transform	PMD	Polarization Mode Dispersion
EDFA	Erbium-Doped Fiber Amplifier	PSK	Phase Shift Keying
ER	Eigenvalue Removal	RRC	Root Raised Cosine
FT	Fourier Transform	SDM	Space Division Multiplexing
INFT	Inverse Nonlinear Fourier Transform	SE	Spectral Efficiency
MI	Mutual Information	WDM	Wavelength-Division Multiplexing
MSSI	Mid-Span Spectral Inversion	ZS	Zakharov-Shabat (System)
NFDM	Nonlinear Frequency Division Multiplexing		
